# CD4 Cell Count: A Critical Tool in the Human Immunodeficiency Virus Response

**DOI:** 10.1093/cid/ciab658

**Published:** 2021-07-26

**Authors:** Nathan Ford, Tom Chiller

**Affiliations:** 1 Human Immunodeficiency Virus, Hepatitis, and Sexually Transmitted Infections Department, World Health Organization, Geneva, Switzerland; 2 Mycotic Diseases Branch, Division of Foodborne, Waterborne, and Environmental Diseases, Centers for Disease Control and Prevention, Atlanta, Georgia, USA

**(See the Major Article by Yapa et al on pages 1350–9.)**

Reducing illness and death from human immunodeficiency virus (HIV) globally has relied on simplification of care delivery so that treatment can be started safely in as many people as possible. Using a public health approach, care provision has been shifted from physicians to nurses, and care delivery has been decentralized from hospitals to primary care clinics and within the community. Treatment has been simplified from combinations of different pills adjusted for age, pregnancy status, and coinfection to a single 3-in-1 pill that is safe and effective for almost everyone. The decision of when to start treatment has evolved from treating the sickest to treating everyone as soon as possible after diagnosis [1].

This public health approach has supported an impressive increase in the number of people receiving antiretroviral therapy, with 27.4 million people living with HIV on treatment in 2020, up from just 7.8 million in 2010. AIDS-related deaths have fallen by 43% since 2010, to 690 000 in 2020 [2].

A study from South Africa in this issue of *Clinical Infectious Diseases* [3] reports an increase in health status of people living with HIV, as measured by CD4 cell count at start of treatment, after the adoption of a universal test and treat policy in 2016. This study used an interrupted time series analysis—a quasi-experimental design used to assess the impact of an intervention or exposure when randomization is not possible or appropriate; interrupted time series analysis is a particularly useful approach for assessing the “real-world” effect of a health policy change [4–6].

The study found that while CD4 cell count at the start of antiretroviral therapy (ART) increased immediately after implementation of the universal test and treat policy in South Africa, the long-term effects were modest. Importantly, an increasing proportion of ART initiators did not have a baseline CD4 cell count and, among those who did, a large proportion had advanced HIV disease (defined as a CD4 cell count <200/μL). These 2 findings—a decrease in CD4 count being obtained at baseline, and a persistence of advanced HIV disease despite ART scale-up—have been reported by other studies [7, 8].

Laboratory testing is one aspect of the public health approach to HIV care delivery that has been questioned since it was first put forward 15 years ago [9]. In high-income settings, diagnostic tests are used to measure immunological and clinical status (CD4 cell count), virological response (viral load), drug resistance (genotyping), and toxicity monitoring. In low- and middle-income settings the relative importance of various laboratory tests has been seen as a trade-off against resources that could be directed instead at scaling up ART. For genotyping and toxicity monitoring, the consensus has been that these tests are not needed as part of routine care, provided that safe and effective treatment can be ensured.[1, 9] Viral load was initially considered too technologically complex for low- and middle-income settings, but major investment has been made to improve access to viral load, with viral suppression now recognized as major indicator of program success [10].

Measuring CD4 cell count was the first laboratory test used for HIV patient care and has been essential to monitoring the clinical risk of opportunistic disease and death. More recently, however, it has been one of the most contentious laboratory tests in the HIV response. This is due to that fact that CD4 cell counts have been used to support a range of clinical decisions—when to start ART [11], which medication to use [12], and how to predict viral suppression [13]—and much less often to decide when opportunistic infection diagnostics and prophylactics [14–16] should be administered. The more recent monitoring uses have fallen away as practice has changed with new evidence, better drugs, and the availability of better tools to monitor treatment. This has contributed to a view that CD4 cell count testing is no longer needed.

As highlighted by the study from South Africa [3] and other studies in recent years [17–21], HIV programs are still challenged to provide care for people with advanced HIV disease—either because the disease is diagnosed late in its progression or because people disengage from care and present again to care after a period without treatment and with a low CD4 cell count.

The advanced HIV disease package of care recommended by the World Health Organization [22] includes diagnostic and prophylactic interventions to respond to the leading causes of disease and death among people living with HIV: tuberculosis, cryptococcal meningitis, and severe bacterial infections [23]. This approach is based on the results of 2 randomized trials [24, 25], each of which found a near-30% reduction in mortality rate associated with the provision of a simple package of interventions for people presenting with advanced HIV disease. Both of these trials relied on CD4 cell count to identify patients who would benefit from receiving the intervention package.

Provision of the advanced HIV disease package of care is part of the public health response to HIV to reduce mortality rates associated with advanced HIV disease. A CD4 cell count is needed to identify people who should receive the package of care, and for this purpose alone it remains a critical tool in the HIV response.

## Note

***Potential conflicts of interest.*** The authors: No reported conflicts of interest. Both authors have submitted the ICMJE Form for Disclosure of Potential Conflicts of Interest. Conflicts that the editors consider relevant to the content of the manuscript have been disclosed.
